# Isolation, structure characteristics and antioxidant activity of two water-soluble polysaccharides from *Lenzites betulina*

**DOI:** 10.1186/s13065-021-00741-6

**Published:** 2021-03-17

**Authors:** Lei Guo, Hongwei Dai, Jiayu Ma, Junmin Wang, Yan Hua, Lingyun Zhou

**Affiliations:** 1 Key Laboratory for Forest Resources Conservation and Utilization in the Southwest Mountains of China, Ministry of Education, Southwest Forestry University, Kunming, People’s Republic of China; 2 School of Life Science, Southwest Forestry University, Kunming, People’s Republic of China; 3 School of Pharmacy, Wannan Medical College, Wuhu, People’s Republic of China

**Keywords:** *Lenzites betulina*, Polysaccharides, Deproteinization, Purification, Structure characteristics, Antioxidant activity

## Abstract

**Background:**

Fungal polysaccharides belong to a very important class of biological macromolecules in nature, and have complex monosaccharide composition and structure. These studies on structure and biological activity of fungal polysaccharides have become one of the research hotspots of scholars at home and abroad.

**Results:**

This study was performed in order to understand the structural characteristics and antioxidant activity of polysaccharides from *Lenzites betulina* (LBPs). The LBPs were deproteinized using sevag method, and further purified by DEAE cellulose-52 column and Sephadex G-100 column chromatographies, then the two refined polysaccharides were obtained and named LBPs-5 and LBPs-6. Fourier transform infrared spectrometry (FT-IR) showed that LBPs-5 and LBPs-6 are typical β-pyranose with characteristic peaks of polysaccharides. The molecular weight of the two water-soluble polysaccharides were estimated to be 3.235 × 10^3^ Da and 6.196 × 10^3^ Da by HPGPC, respectively. HPLC with PMP derivatization analysis indicated that the monosaccharide compositions of LBPs-5 were mannose, glucuronic acid, glucose, and galactose in a molar ratio of 0.05:0.15:0.76:0.04. The monosaccharide compositions of LBPs-6 were mannose, glucuronic acid, and glucose, in a molar ratio of 0.04:0.17:0.79. Furthermore, the two water-soluble polysaccharides demonstrated strong scavenging effects on DPPH·, ABTS·^+^, ·OH and weak total reducing power, especially LBPs-6 was significantly stronger in scavenging rate than that of LBPs-5.

**Conclusions:**

The outcome of the study indicated that LBPs had good potential as medicine and food.

## Introduction

Natural plant polysaccharides are a very important class of biological macromolecules in plants, which are composed of aldoses and ketoses through glycosidic bond, and are the basic substances that effectively maintain and ensure the normal operation of biological life activities [1, 2]. Fungal polysaccharides have a variety of physiological activities and are widely used in medicine, agriculture, food and other industries [3–5]. They have various physiological functions such as immune regulation, antitumor, antiviral and antioxidant [6–9]. They can be used as natural immunomodulator. These studies have become one of the research hotspots of scholars at home and abroad. The chemical structure of fungal polysaccharides plays an important role in their biological activities, including their molecular configuration, molecular weight, branched chains, branched groups and main chains [10].

*Lenzites betulina* (L.) Fr. is a medicinal mushroom and widely distributed in China, which is a *Lenzites* fungus belonging to the Polyporacea [11, 12]. Because of containing some physiologically active substances, such as polysaccharides, pyranone, sterol et al. *Lenzites betulina* has been used to treat haunch and femora pain, acropathy, apoplexy and cold [13–16]. However, little is known about the molecular structure and physiological function of polysaccharides from *Lenzites betulina*. Therefore, in this study, the crude polysaccharides from *Lenzites betulina* (LBPs) was deproteinized using sevag method, and purified sequentially by DEAE cellulose-52 column and Sephadex G-100 column chromatographies, then two refined polysaccharides were obtained. Moreover, their structure characteristics, relative molecular weights (Mw) and monosaccharide composition were determined. To the best of our knowledge, this is the first study to investigate the structural characteristics of polysaccharides from *Lenzites betulina.* It will lay a foundation for further understanding the biological activity mechanism of LBPs.

## Results and discussion

### Deproteinization of LBPs

**Fig. 1:**
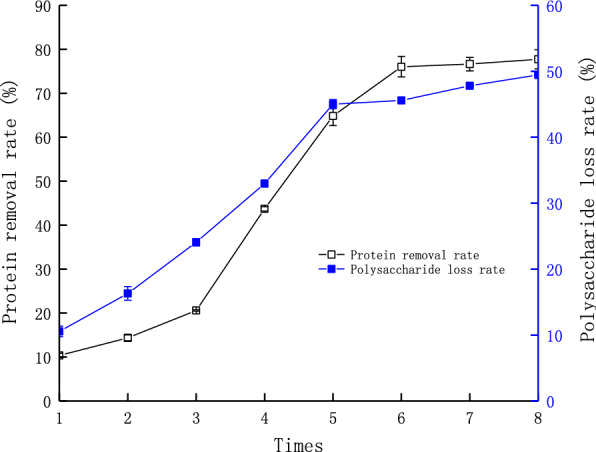
Effects of experiments times on protein removal rate and polysaccharide loss rate

The protein removal rate and polysaccharide loss rate could be seen in Fig. 1. When the protein was removed, polysaccharides were partially removed at the same time. It was obvious that both protein and polysaccharides removal rate increased with increasing experiment times. The protein removal rate reached the maximum of 76.04% when the experimental was repeated 6 times, and the polysaccharides loss rate reached 45.58%. After 6 times, the two indexes reached to a dynamic equilibrium with the experimental times increasing. Therefore, an experimental time of 6 was chosen for the further experiments.

After deproteinization, the crude LBPs were concentrated and freeze-dried, and then the LBPs sample was obtained for further structure identification. The sample were resolved in distilled water and then scanned in the range of 200–400 nm by an ultraviolet spectrophotometer. The results of UV analysis showed that there was not absorption peak at 260 nm and 280 nm, and it could proved that no protein or nucleic acid in the sample.

### Purification by DEAE cellulose-52 column

**Fig. 2:**
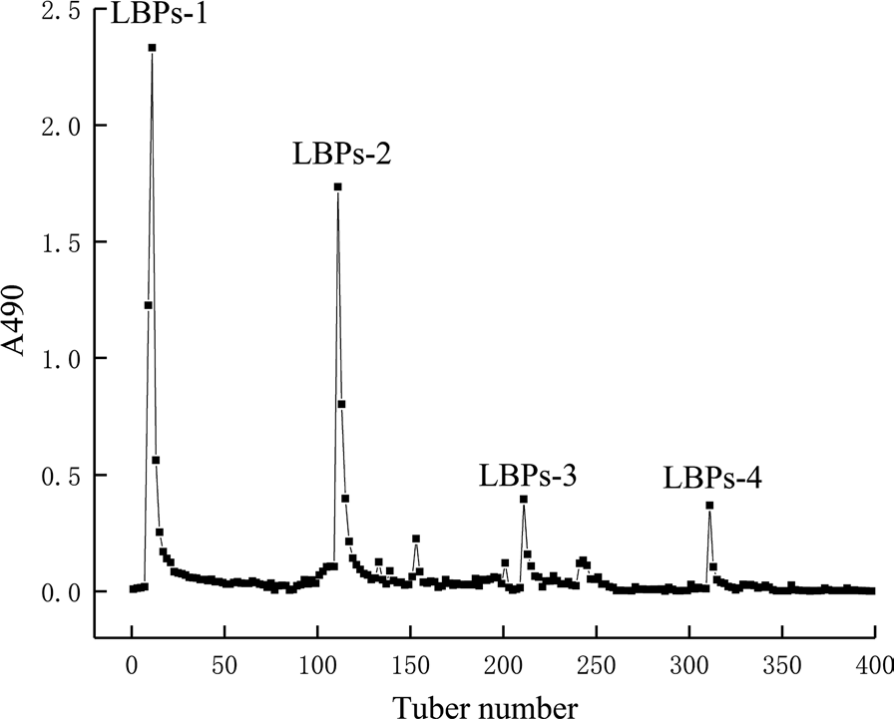
Gradient elution profile of polysaccharides extracted from *Lenzites betulina* by DEAE cellulose-52 chromatography

Purification of LBPs was performed by DEAE cellulose-52 column, and they were eluted with gradients of 0, 0.1, 0.2 and 0.5 mol/L NaCl solutions. The fractions of the isolated LBPs were mainly concentrated in the elution solution. As shown in Fig. 2, four polysaccharides components were separated and obtained from LBPs. Among these, the content of LBPs-1 and LBPs-2 were higher than the other, so the two polysaccharides components were selected and further purified by the Sephadex G-100 column.

### Further purification by Sephadex G-100 column

**Fig. 3:**
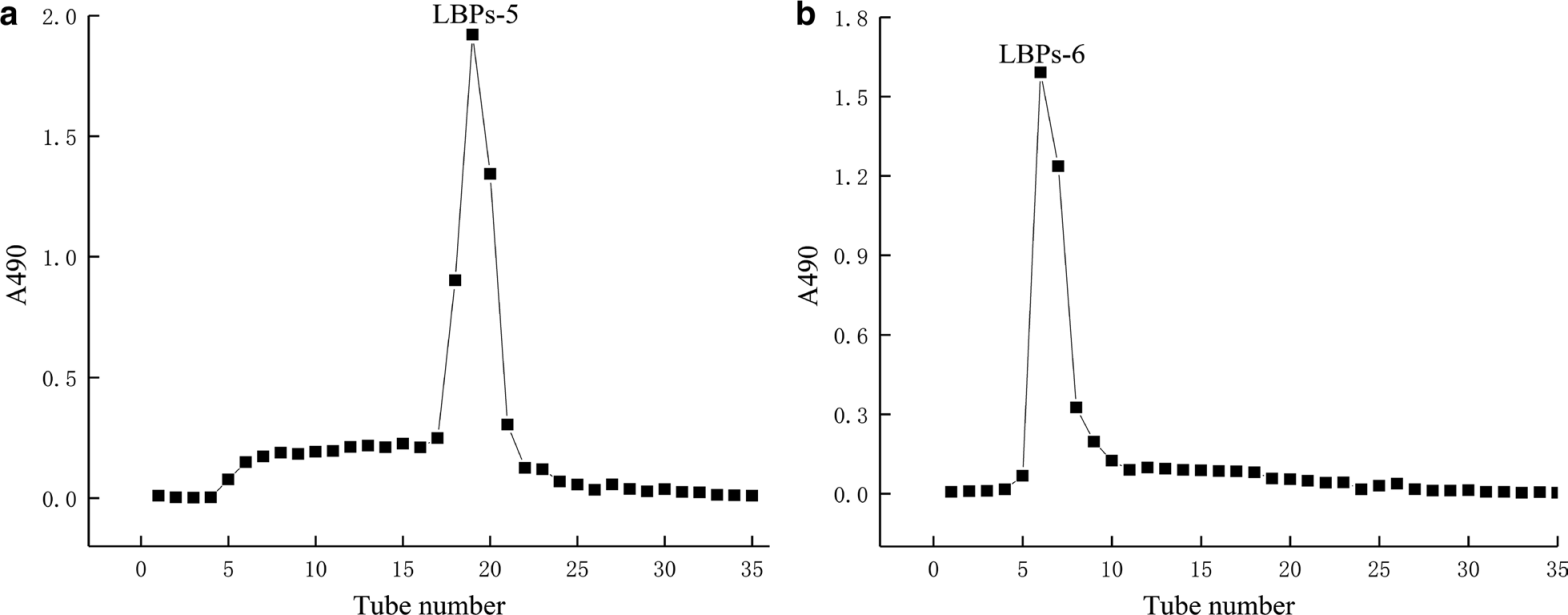
Elution profile of polysaccharides LBPs-5 and LBPs-6 by G-100 column chromatography

The two water-soluble polysaccharides of LBPs-1 and LBPs-2 were further purified using a Sephadex G-100 column, and the elution curve of them were shown in Fig. 3. After purification by G-100, two single eluting symmetrical peaks were obtained. The results indicated that the obtained polysaccharide of LBPs-5 and LBPs-6 were a relatively homogeneous polysaccharide.

### Fourier transform infrared (FT-IR) analysis

**Fig. 4:**
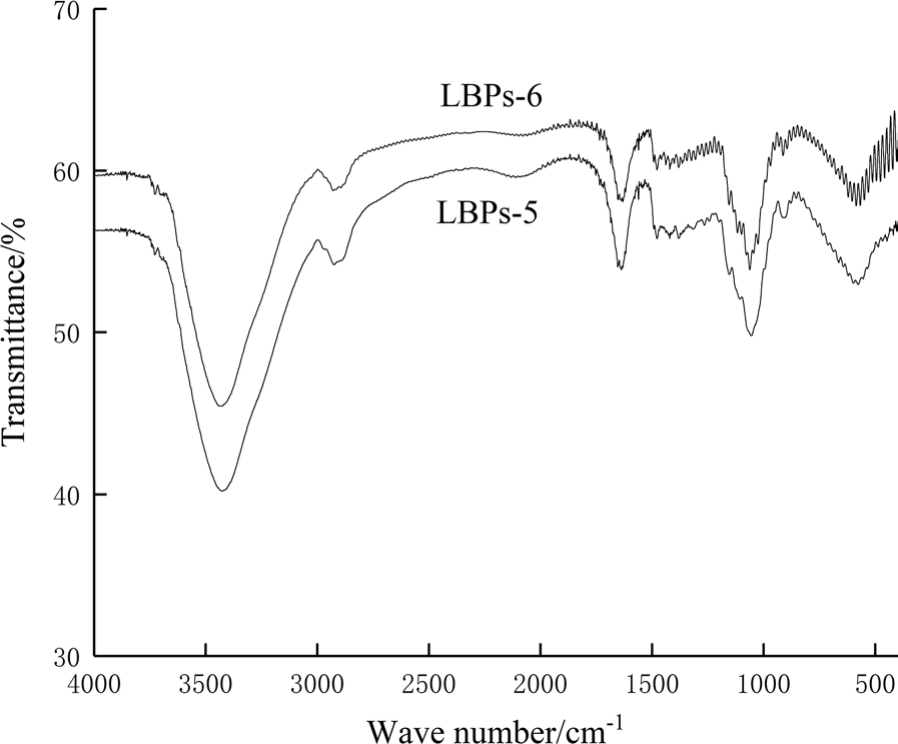
The FT-IR spectrums of LBPs-5 and LBPs-6

Figure 4 showed the Fourier transform infrared (FT-IR) spectra of dialyzed LBPs-3 and LBPs-4, which had virtually identical characteristic absorption peaks. The strong and broad peak at around 3424 cm^−1^ was assigned to (OH–) stretching of polysaccharide residues and residual water (OH–). The signal at 2925 cm^−1^ was attributed to the asymmetric stretching vibrations of (C–H) groups of polysaccharides [17]. The bands at ~ 1637 cm^−1^ (LBPs-5), 1752 cm^−1^ (LBPs-6), 1546 cm^−1^ (LBPs-5), 1652 cm^−1^ (LBPs-6) were attributed to stretching vibration of C=O [18, 19]. The weak peaks observed at 1420 cm^−1^ (LBPs-5), 1478 cm^−1^ (LBPs-6) indicated the stretching vibration of C–H [20]. Peaks observed at 1054 cm^−1^ (LBPs-5) and 1078 cm^−1^ (LBPs-6) could be assigned to the contribution of C–O–C symmetric stretching vibration [21]. Sharp absorption peaks at 915 cm^−1^ and 912 cm^−1^ suggested the presence of β-glucosidic bond in the molecular structure of LBPs-5 and LBPs-6 [22, 23]. Weak absorption peaks at 554 cm^−1^ and 585 cm^−1^ suggested the characteristics of pyranoid ring in the molecular structure of LBPs-5 and LBPs-6 [21]. Overall, the outcomes of FT-IR spectra showed all typical absorption peaks associated with polysaccharides which confirmed the identity of LBPs-5 and LBPs-6 as polysaccharides.

### Molecular weight determination

The molecular weight of LBPs-5 and LBPs-6 were determined using high performance gel permeation chromatography (HPGPC). According to the regression equation (log _Mw_ = − 1.7568 X + 2.334, R^2^ = 0.9976), the retention time of the LBPs-5 component was 22.922 min. the Mw of LBPs-5 was approximately calculated as 3.235 × 10^3^ Da. The retention time of LBPs-6 component was 22.108 min, and the Mw calculated on the basis of the regression equation was 6.196 × 10^3^ Da.

### Monosaccharide composition

**Fig. 5:**
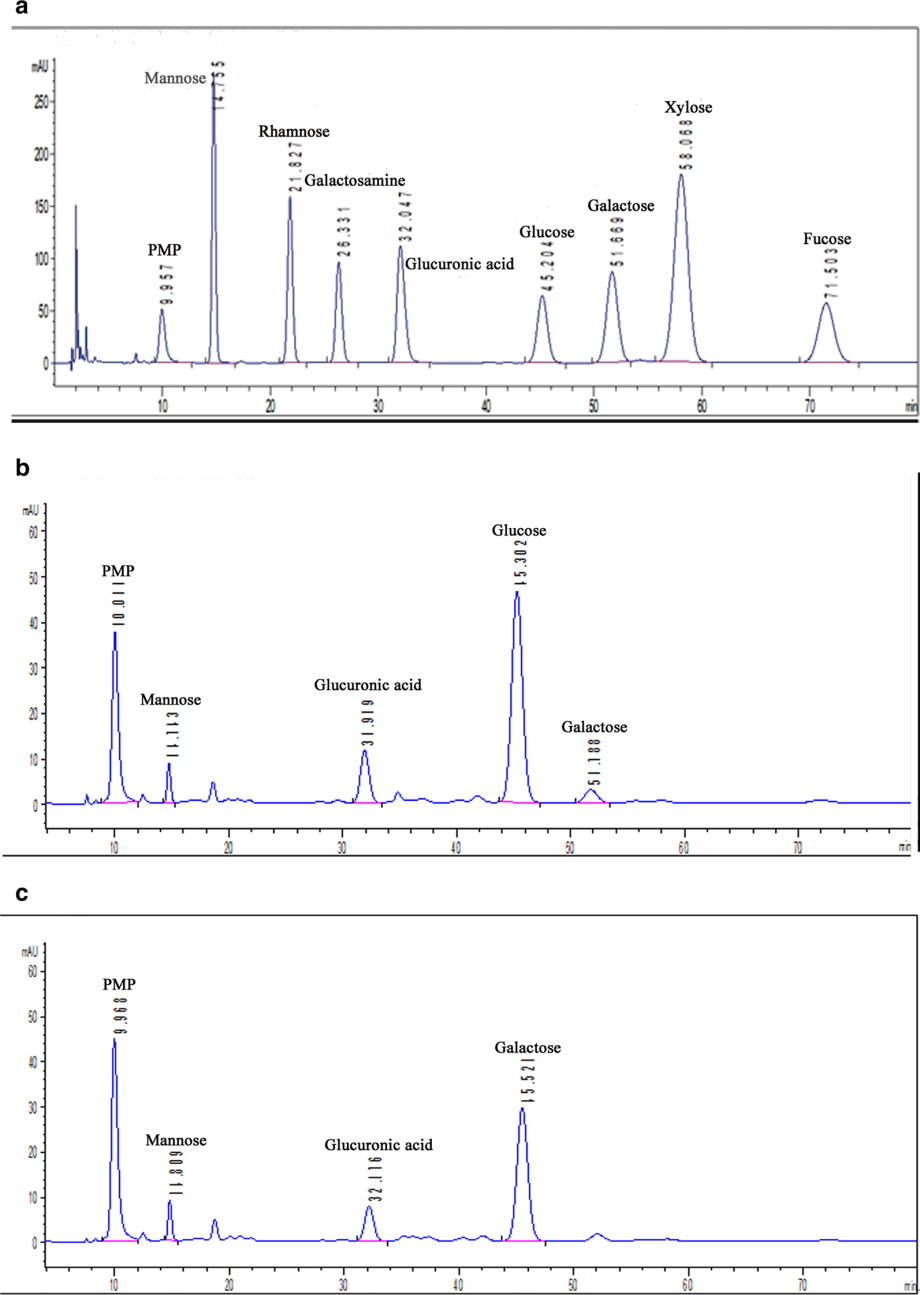
HPLC spectra of monosaccharide compositions of Standard sample (**a**), LBPs-5 (**b**) and LBPs-6 (**c**)

The HPLC chromatograms of the PMP-derivatized component monosaccharides released from the polysaccharides fractions are shown in Fig. 5. The monosaccharide compositions of LBPs-5 were consisted of mannose, glucuronic acid, glucose, and galactose in a molar ratio of 0.05:0.15:0.76:0.04 (Fig. 5a). LBPs-6 was mainly composed of mannose, glucuronic acid, and glucose in a molar ratio of 0.04:0.17:0.79 (Fig. 5b). According to the results of the monosaccharide composition the two polysaccharides are heteroglucans.

### Antioxidant activities of the two water-soluble polysaccharides

**Fig. 6:**
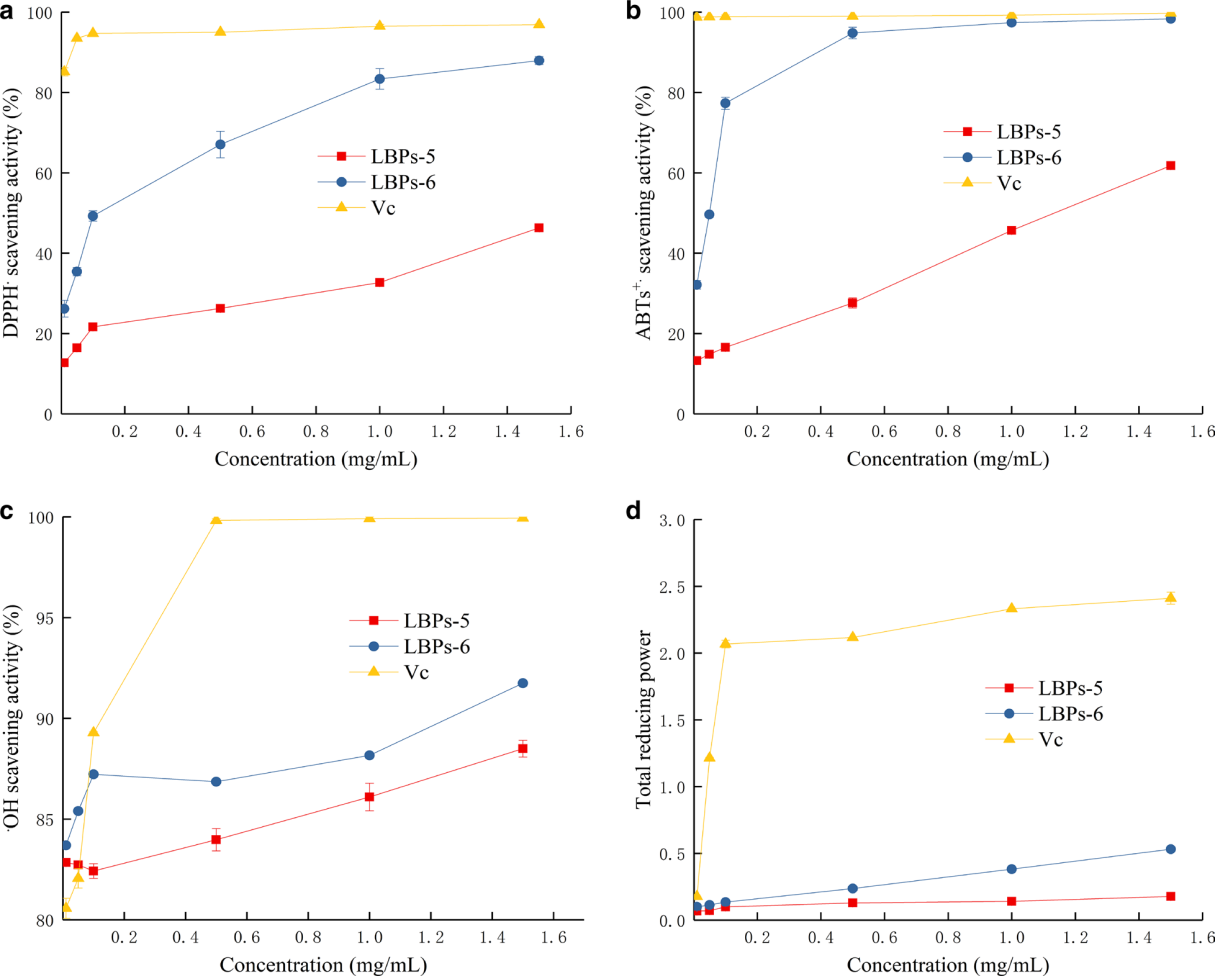
Scavenging activities on DPPH· (**a**), ABTS^.+^ (**b**), ·OH (**c**), and total reducing power (**d**) assay for LBPs-5, LBPs-6 and Vc at different concentrations

The assay of the scavenging of DPPH radical (DPPH·) is a widely applied method to evaluate the antioxidant activity of natural active ingredients from different plant origin [24]. Polysaccharides have many hydroxyl groups and most of them can donate hydrogen to reduce the DPPH· [25]. The DPPH· scavenging activity of the two water-soluble polysaccharides and Vc were shown in Fig. 6a. The results showed that LBPs-5 and LBPs-6 exhibited an inhibitory activity against DPPH· in a dose-dependent manner. The DPPH· scavenging activity of LBPs-5 and LBPs-6 increased steadily at the concentration rang of 0.01–1.5 mg/mL, while Vc displayed a maximum plateau from 0.01 to 0.1 mg/mL, indicating that the scavenging activity of the two water-soluble polysaccharides against DPPH· were less than that of Vc at the same concentrations. The DPPH· scavenging activity of LBPs-5 and LBPs-6 were 46.31 ± 0.71% and 87.96 ± 0.97% at the concentration of 1.5 mg/mL (p < 0.05). As shown in Table 1, the EC_50_ value of LBPs-5 and LBPs-6 were 1.66 mg/mL and 0.095 mg/mL, while those of Vc was 0.009 mg/mL.

**Table 1:** EC_50_ values of various sample from *Boletus edulis* in antioxidant properties

Sample	EC_50_ value (mg/mL)		
	DPPH· scavenging activity	ABTS^.+^ scavenging activity	·OH scavenging activity
LBPs-5	1.66^c^	1.369^c^	0.006^a^
LBPs-6	0.095^b^	0.031^b^	0.006^a^
Vc	0.009^a^	0.004^a^	0.010^b^

EC_50_ value is half maximal inhibitory concentration. Data were presented as the mean value (n = 3). The superscript letters a, b, and c indicate a significant difference at the 0.05 significance level

ABTS can generate turquoise and water soluble ABTS free radicals (ABTS^.+^) after oxidized by potassium sulfate, and the characteristic absorption peak of which can be detected at 734 nm. Fungal polysaccharides can scavenge ABTS^.+^ and further makes the turquoise solution fading, and the ABTS^.+^ scavenging activity could be measured according to the fading degree [26]. Figure 6b demonstrated the ABTS^.+^ scavenging activity of the two water soluble polysaccharides and Vc. These findings indicated that LBPs-5 exhibited higher ABTS^.+^ scavenging activity than LBPs-6 at all tested concentrations. The scavenging effects of three samples on the ABTS^.+^ followed the order: Vc > LBPs-6 > LBPs-5 and were 99.75 ± 0.25%, 98.32 ± 0.38%, and 61.80 ± 0.38% at the concentration of 1.5 mg/mL (p < 0.05), respectively. As shown in Table 1, the EC_50_ values corresponding to the ABTS^.+^ scavenging activity were found to be 1.369 mg/mL, 0.031 mg/mL, and 0.004 mg/mL for the original LBPs-5, LBPs-6, and Vc, respectively.

Hydroxyl radicals (·OH) could be byproducts of immune action, which are highly reactive and can damage various macromolecules, such as carbohydrates, lipids and amino acids and are very harmful to human health [27]. The production of ·OH is dependent on the content of Fe^2+^ and H_2_O_2_ according to the Fenton reaction [28]. The scavenging effects of LBPs-6 and LBPs-5 on ·OH were shown in Fig. 6c. LBPs-5 and LBPs-6 demonstrated stronger ·OH scavenging activities than Vc at concentrations from 0.01 to 0.05 mg/mL (p < 0.05). At a concentration of 0.01 mg/mL, the ·OH scavenging activity increased in the order of Vc < LBPs-5 < LBPs-6 and were 80.57 ± 0.50%, 82.85 ± 0.05%, and 83.70 ± 0.09%, respectively. Table 1 demonstrated the EC_50_ values of scavenging ·OH of various samples, and the findings indicated that LBPs-5 and LBPs-6 were observed to posses highly ·OH scavenging potential than that of Vc.

The total reducing power is generally associated with the presence of reductones, which could donate a hydrogen atom and exert antioxidant action by breaking the free radical chain [29]. The total reducing power of an active compound could serve as a important indicator of its potential antioxidant activity. The total reducing power of all three samples were measured at 700 nm and shown in Fig. 6d. The total reducing power of LBPs-6 and LBPs-5 increased with increasing concentrations. The total reducing power increased from 0.1 to 0.53 when the concentration of LBPs-6 changed from 0.01 mg/mL to 1.5 mg/mL. In addition, the total reducing power of LBPs-5 was lower than that of LBPs-6 at the same concentration, it reached 0.177 at a concentration of 1.5 mg/mL.

The antioxidant tests indicated that both of LBPs-5 and LBPs-6 have demonstrated the good scavenging activities on the three radicals (DPPH·, ABTS^.+^, and ·OH) and the total reducing power, especially LBPs-6 was slightly stronger in antioxidant activity than that of LBPs-5. In general, the antioxidant activities of polysaccharides are highly associated with the glucuronic acid content. Wu et al. [30] reported that the antioxidant activity increased with the increase of glucuronic acid content, and the molecular weight of polysaccharides on its antioxidant activity was not significant. This is further supported by Wu et al. who proposed that the high glucuronic acid content of polysaccharides could be one of the main contributors to the antioxidant activity of *Leccinum rugosiceps* [31].

## Materials and methods

### Materials and regents

*Lenzites betulina* fruiting bodies were purchased in April 2017 from Shenzhen Yueyun Trading Co., Ltd., and were identified by associate professor ZHAO Changlin of Southwest Forestry University. A voucher specimen was deposited with the Forestry College of Southwest Forestry University. Cellulose DEAE-52 was purchased from Shanghai Goldwheat biotechnology co., LTD (Shanghai, China). Sephadex G-100 was purchased from shanghai Baoman Biotechnology co. LTD (Shanghai, China). All monosaccharide standards (mannose, rhamnose, galactosamine, glucuronic acid, glucose, galactose, xylose, arabinose, fucose) were purchased from Sigma Chemical Co., Ltd. (St. Louis, MO, USA). All other chemicals reagents were of analytical grade.

### Extraction of crude LBPs

Firstly, dried sample was crushed and then decolorized using 10-fold volume of ethanol (80%) at 60 ℃ for 30 min twice by ultrasonic cleaner (SB25-12DTDS, Ningbo Xinyi Ultrasonic equipment co., Ltd), and the organic layer was removed by suction filtration. The residue was extracted under the conditions of cellulase dosage of 0.8%, enzymolysis temperature of 60 °C, enzymolysis time of 180 min, pH of 4.5, liquid–solid ratio of 45 ml/g, ultrasound power of 300 W, ultrasound time of 20 min and ultrasound temperature of 45 °C, respectively [32]. Secondly, the supernatant were concentrated and then precipitated with a four-fold volume of anhydrous ethanol at 4 ℃ overnight. Then the precipitates were collected by centrifugation at 5000 rpm for 15 min. At last, the crude LBPs was obtained by freeze-drying for 24 h.

### Deproteinization of crude LBPs

Protein in the crude LBPs could has a great interference effect to analysis the structural and biological activity of polysaccharides. In general, deproteinization of polysaccharides was carried out Sevag method [33], briefly, the Sevag solution (chloroform and *n*-butanol, 4:1, v/v) is added in the crude LBPs solution (1 mg/mL). The mixed solution was shocked for 30 min each time, then protein and organic reagent was removed by centrifugation. The content of protein and polysaccharides in the crude LBPs were determined according to coomassie bright blue method and phonel-sulfate method [34]. The remove rate of protein in LBPs was calculated by a bovine serum albumin standard curve, and it was presented as *A* = 6.739 *C*—0.0033 (where *A* is the absorbance value of sample, *C* (mg/mL) is the concentration of bovine serum albumin solution, *R*^*2*^ = 0.9998). The loss rate of polysaccharides in LBPs was calculated by a glucose standard curve and it was presented as A = 0.016 C + 0.0071 (where *A* is the absorbance value of sample, *C* (mg/mL) is the concentration of glucose solution, *R*^*2*^ = 0.9993). After deproteinization for six times, crude LBPs was obtained by concentrated and freeze dried.

### Purification of crude LBPs by DEAE cellulose-52 column

Crude LBPs (30 mg) was dissolved in deionized water (3 mL), and the solution was slowly added to the well-balanced DEAE-52 column using a dropper along the column wall. Gradient elution was sequentially performed with 0, 0.1, 0.2 and 0.5 mol/L NaCl solutions at a flow rate of 1 mL/min, and 100 tubes were collected per gradient for 10 min each. The phenol-sulfate method was used to detect the polysaccharide content, and the elution curve was plotted according to the content [25]. The two samples with higher polysaccharide content was collected, concentrated and lyophilized to obtain LBPs-1 and LBPs-2. The salt was removed by dialysis and then lyophilized.

### Further purification by Sephadex G-100 column

The two polysaccharide fraction obtained in the previous step was mixed with 10 mL of a 3 mg/mL solution in deionized water and slowly added to the equilibrated Sephadex G-100 column. Elution was performed with deionized water at a rate of 0.3 mL/min, and elution fractions were automatically collected, 10 mL per tube. The phenol-sulfate acid method was used to detect the absorbance of the collected samples and to draw the elution gradient curve [25]. The sample with the highest polysaccharide content was selected and collected by a rotary evaporator, concentrated, dialyzed, desalted and lyophilized to obtain pure LBPs-5 and LBPs-6.

### FT-IR analysis

FT-IR spectra of LBPs-5 and LBPs-6 were analyzed using a FT-IR spectrophotometer (spectral resolution of 1 cm^−1^, scanned for 32 times) in the range of 400–4000 cm^−1^. Approximately 1 mg samples were mixed with 100 mg dried KBr powder and then pressed into pellets for the FT-IR measurement [35].

### Analysis of monosaccharide composition of LBPs-5 and LBPs-6

The monosaccharide composition of LBPs-5 and LBPs-6 were determined using the high-performance liquid chromatography (HPLC) analysis of monosaccharide derivatives with 1-phenyl-3-methyl-5-pyrazolone (PMP) according to the method of Li et al. [36]. In brief, the two polysaccharide sample (2 mg) and monosaccharide standard were added 1 mL trifluoroacetic acid (TFA, 2 M) to hydrolyze at 110 ℃ for 4 h, respectively. After hydrolysis, methanol solution was added and then evaporated to remove excess TFA. The PMP derivatization method was as follows: 50 μL hydrolysate of the different sample was pipetted into 50 μL NaOH solution (0.6 M) and mixed well, respectively. Then the mixture was added in 100 μL of methanol solution of PMP (0.5 M). The following reaction took place in a constant temperature drying oven at 70 ℃ for 30 min, and then removed it and cooled down. Next, 100 μL hydrochloric acid (0.6 M) was added for neutralization, and the mixture was extracted three times with chloroform. The aqueous layer was filtered through a 0.22 μm membrane and analyzed by HPLC. The samples (20 μL) were injected into an Agilent system (Agilent Technologies Inc. USA) equipped with a C18 column (150 m × 4.6 mm × 5 μm). Acetate Buffer (0.1 M)-acetonitrile (83:17, V/V) was used as the mobile phase at a flow rate of 1.0 mL/min, and the column oven temperature was 30 °C.

### Determination of the molecular weight of LBPs-3 and LBPs-4

High-performance gel permeation chromatography (HPGPC) was used to measure the molecular weight of the polysaccharide fraction after two stepwise purifications of DEAE-52 and G-100 [37]. The molecular weight of the two water-soluble polysaccharides (LBPs-5 and LBPs-6) were evaluated using a HPGPC system (Agilent 1260 HPLC system) equipped with a Shodex Ohpak SB-806 HQ gel column and differential detector. In addition, 0.1 M sodium chloride solution was used as the mobile phase, the flow rate was 0.5 mL/min, and the column temperature was 35 ℃. At the same time, the glucan standards whose molecular weight range was close to that of the samples were selected (the molecular weight was 2.5, 4.6, 7.1, 10, 21.4, 41.1, 84.4 and 133.8 kDa) as a standard curve, according to the standard curve to obtain a linear regression equation as follows: y = − 1.7568x + 2.334 (R^2^ = 0.9976); where y is the logarithm of the molecular weight of the polysaccharide; x is the retention time of the glucan standards (min). After this, 20 μL of the standard and sample were injected, and the data were analyzed using Agilent GPC software. Based on the retention time of the polysaccharide on the column and the standard glucan molecular weight curve of the column, the molecular weight of LBPs-5 and LBPs-6 were calculated.

### Antioxidant activity assays

The two water-soluble polysaccharides (LBPs-5 and LBPs-6) were prepared above was diluted with distilled water into different concentrations of 0.01, 0.05, 0.1, 0.5 1.0 and 1.5 mg/mL. The antioxidant activity was investigated by scavenging activity of DPPH·, ABTS^.+^, ·OH and total reducing power. A positive control of Vc was used.

#### Scavenging effect on DPPH·

The DPPH· scavenging activity was determined according the method of Wang et al. [38] with slight modifications. Briefly, 2 mL of 0.2 mM DPPH dissolved in absolute ethyl alcohol was added to 2 mL of sample. Then the mixture was kept in the dark for 30 min at room temperature, and the absorbance was recorded at 517 nm. The DPPH· scavenging activity was calculated as follows:

$$\begin{aligned} & {\text{DPPH}}\cdot{\text{ scavenging activity }}\left( \% \right) \, \\ &\quad= \, \left[ {1 - \, \left( {A_{1} - \, A_{2} } \right) \, /A_{0} } \right] \, \times \, 100, \end{aligned}$$

where A_1_ is the absorbance value of sample, A_2_ is the background absorbance value (absolute ethyl instead of DPPH solution), and A_0_ is the absorbance value of the blank (absolute ethyl instead of the sample).

#### Scavenging effect on ABTS^.+^

The scavenging activity of ABTS^.+^ was carried out according to the previous method [39]. In ordering to generate ABTS^.+^, an equal volume of 7 mM ABTS and 2.45 mM potassium persulfate solutions were mixed and allowed to stand in the dark at room temperature overnight. Then the mixture was diluted with absolute ethyl alcohol, and used for the assay. 2 mL of the sample was added into 2 mL of the mixture, after 5 min of incubation in the dark at room temperature. The absorbance value was measured at 734 nm and the ABTS^.+^ scavenging activity was determined by the following equation:

$$\begin{aligned}& {\text{ABTS}}^{. + } {\text{scavenging activity }}\left( \% \right) \, \\ &\quad= \, \left[ {1 - \, \left( {A_{1} - \, A_{2} } \right) \, /A_{0} } \right] \, \times \, 100, \end{aligned}$$

where A_1_ is the absorbance value of sample, A_2_ is the background absorbance value (absolute ethyl instead of the mixture), and A_0_ is the absorbance value of the blank (absolute ethyl instead of the sample).

#### Scavenging effect on ·OH

The ·OH scavenging activity was measured according to a reported method described previously [40] with little modifications. In briefly, 0.2 mL of ferrous sulfate (3 mM) and 0.2 mL of salicylic acid–ethanol (6 mM) were added to 1 mL of sample solution, 0.2 mL of hydrogen peroxide (4 mM) was added finally to start the reactions, then incubated at room temperature for 60 min. The absorbance value was calculated at 510 nm. The ·OH scavenging activity was calculated using the following formula:

$$\begin{aligned}& {\text{ABTS}}^{. + } {\text{scavenging activity }}\left( \% \right) \, \\ &\quad= \, \left[ {1 - \, \left( {A_{1} - \, A_{2} } \right) \, /A_{0} } \right] \, \times \, 100, \end{aligned}$$

where A_1_ is the absorbance value of sample, A_2_ is the background absorbance value (distilled water instead of hydrogen peroxide), and A_0_ is the absorbance value of the blank (absolute ethyl instead of the sample).

#### Total reducing power assay

The total reducing power of polysaccharides was measured by the method of Li [41] with some modifications. A volume of 1 mL of sample solution was mixed 2.5 mL phosphate buffer (pH 6.6, 0.2 M) and 2.5 mL of potassium ferricyanide solution (1%, W/V), and then the mixture was incubated at 50 ℃ for 20 min. Then 2.5 mL trichloroacetic acid (10%, W/V) was added into the mixture and centrifuged at 3500 rpm for 10 min. 2.5 mL of the supernatant was mixed with 2.5 mL of distilled water and 0.5 mL of ferric chloride solution (0.1%, W/V) at room temperature for 15 min. The absorbance value of the reaction mixture was measured at 700 nm.

### Statistical analysis

All experiments were repeated at least three times, statistical analysis was carried out using Originpro 2017C (OriginLab Co., USA) and SPSS 20.0.0 statistics software (IBM Co., USA). All values were presented as the mean ± SD, multi-group mean comparison was analyzed by one-way variance, and pairwise comparison was performed by LSD test. Significance difference was set at *p* < 0.05.

## Conclusions

In this study, two water-soluble polysaccharides LBPs-5 and LBPs-6 were isolated from *Lenzites betulina* using cellulase-ultrasonic synergistic extraction method, successfully purified via DEAE cellulose-52 and sephadex G-100 column and characterized by a combination of chemical and instrumental analysis methods. The strong antioxidant activities indicates the two water-soluble polysaccharides have potential as a source of natural antioxidants with potential application in reducing oxidative stress with consequent health benefits. Anyway, the antioxidant mechanism of the two water-soluble polysaccharides is still not clear and thus further work is required to clarify the possible antioxidant and antitumor mechanism and of *Lenzites betulina* polysaccharides.

## Data Availability

The datasets used and/or analysed during the current study are available from the corresponding author on reasonable request.

## References

[1] Song JB, Chen M, Li ZQ, Zhang JF, Hu H, Tong XL, Dai FY (2019). Astragalus polysaccharide extends lifespan via mitigating endoplasmic reticulum stress in the silkworm, *Bombyx mori*. Aging Dis.

[2] Wu MQ, Xia W, Xu ZZ, Song JX, Chen LN, Zhang WQ (2019). Review on isolation and purification, structural elucidation and biological activity of botanical polysaccharides. Chem world.

[3] Liang X, Ni XZ, Han LP (2018). Advances in bioactivities of polysaccharides from edible medicinal fungi. J Changchun Norm Uni.

[4] Janjušević L, Karaman M, Šibul F, Tommonaro G, Lodice D, Pejin B (2017). The lignicolous fungus *Trametes versicolor* (L.) Lloyd (1920): a promising natural source of antiradical and AChE inhibitory agents. J Enzyme Inhib Med Chem.

[5] Pejin B, Tešanović K, Jakovljević D, Kaišarević S, Šibul F, Rašeta M, Karaman M (2017). The polysaccharide extracts from the fungi *Coprinus comatus* and *Coprinellus truncorum* do exhibit AChE inhibitory activity. Nat Prod Res.

[6] Lu HY, Zhang XP (2018). Effect of lentinan on infiltrating lymphocytes of tumor tissue and peripheral blood in breast cancer patients undergoing neoadjuvant chemotherapy. J Anhui Med Pharm.

[7] Gan N, Wu XY, Zheng CJ, Yin H, Wu SL (2017). Study on the antitumor activity of *Auricularia auricula* polysaccharides on B16 melanoma cells. J Guangdong Pharm Univ.

[8] Irinoda K, Masihi KN, Chihara G, Kaneko Y, Katori T (1992). Stimulation of microbicidal host defence mechanisms against aerosol influenza virus infection by lentinan. Int J Immunopharm.

[9] Wei H, Zhang D, Lu L, Chen FC (2017). Extraction, purification and antioxidant activities of several polysaccharides from edible and medicinal fungi. J Nanjing Norm Univ (Nat Sci Ed).

[10] Wang YS, Zheng XX, Li FK, Yang Y, Wang H, Lv SJ (2019). Advances in pharmacological action of polysaccharides from edible-medicinal fungi and its application in veterinary clinic. Chin Anim Husband Veter Med.

[11] Wen CN, Chen HP, Zhao ZZ, Hu DB, Li ZH, Feng T, Liu JK (2017). Two new γ-lactones from the cultures of basidiomycete *Lenzites betulinus*. Phytochem Lett.

[12] Shen XY, Ma T, Ma X, Huang YT, Liu CL (2017). Isolation, purification and free radical scavenging activities of polysaccharide from *Lenzites betulina*. Mycosystema.

[13] Liu K, Wang JL, Liu MF, Bi KL, Song YF (2011). Medical macrofungi resources and diversity in inner Mongolia. J Hebei Univ (Nat Sci Ed).

[14] Fujimoto H, Nakayama M, Nakayama Y, Yamazaki M (1994). Isolation and characterization of immunosuppressive components of three mushrooms, *Pisolithus tinctorius*, *Microporus flabelliformis* and *Lenzites betulina*. Chem Pharmaceut Bull.

[15] Liu K, Wang JL, Wu HB, Wang Q, Bi KL, Song YF (2012). A new pyranone from *Lenzites betulina*. Chem Nat Compd.

[16] Ren G, Liu XY, Zhu HK, Yang SS, Fu CX (2006). Evaluation of cytotoxic activities of some medicinal polypore fungi from China. Fitoterapia.

[17] Li LY, Huang T, Liu HM, Zang JL, Wang P, Jiang XL (2019). Purification, structural characterization and anti-UVB irradiation activity of an extracellular polysaccharide from *Pantoea agglomerans*. Int J Biol Macro.

[18] Boulet JC, Williams P, Doco T (2007). A Fourier transform infrared spectroscopy study of wine polysaccharides. Carbohydr Polym.

[19] Yang YR, Hao ZQ, Chang MC, Meng JL, Liu JY, Feng CP (2019). Isolation, purification, structural identification and antioxidant activity of acidic polysaccharide in *Sparassis crispa*. Acta Edulis Fungi.

[20] Jeong HK, Lee D, Kim HP, Baek SH (2019). Structure analysis and antioxidant activities of an amylopectin-type polysaccharide isolated from dried fruits of *Terminalia chebula*. Carbohydr Polym.

[21] Cai M, Chen S, Luo SL, Yang K, Sun PL (2019). Comparison of membrane separation and alcohol precipitation for the separation of crude polysaccharides from *Hericium erinaceus*. Food Chem.

[22] Liu F, Chen GT, Hu QH, Zhao SW, Zhao LY (2014). Separation, purification and structure characteristics of Zn-binding polysaccharides from *Flammulina velutipes*. Food Chem.

[23] Chen SS, Wu B, Tan T, Xie SS, Yang SL, Feng YS, Wen Q (2019). Isolation, purification and structural characterization of *Bletilla striata* polysaccharides and its antitumor activity. Chin Trad Herbal Drugs.

[24] Brand-Williams W, Cuvelier ME, Berset C (1995). Use of a free radical method to evaluate antioxidant activity. LWT Food Sci Technol.

[25] Benvidi SMH, Jahanbin K (2020). A new water-soluble polysaccharide from *Echinops pungens* Trautv roots. Part I. Isolation, purification, characterization and antioxidant activity. Int J Biol Macromole.

[26] Liu YQ, Hao LM, Lu JK, Cui Y, Zheng ZQ, Zhang LM, Jia SR (2019). Antioxidant activity of *Ganodermalucidum* fruit body and spore powder polysaccharide. Sci Technol Food Ind.

[27] Chen YX, Liu XY, Zheng X, Huang YF, Liu B (2016). Antioxidant activities of polysaccharides obtained from *Chlorella pyrenoidosa* via different ethanol concentrations. Int J Biol Macromole.

[28] Fan LP, Li JW, Deng KQ, Ai LZ (2012). Effects of drying methods on the antioxidant activities of polysaccharides extracted from *Ganoderma lucidum*. Carbohyd Polym.

[29] Gordon MH, Hudson BJF (1990). The mechanism of antioxidant action in vitro. Food antioxidants.

[30] Wu YL, Shao ZL, Zhang Y, Wang YL, Zhao ZM (2019). Isolation, purification and antioxidant activity of pumpkin homogeneous polysaccharide. Food Res Develop.

[31] Wu Y, Xu F, Li XS, Zhu JH, Shen LQ (2020). Structural elucidation and antioxidant activity of LRP-I and LRP-II polysaccharide from *Leccinum rugosiceps*. Sci Technol Food Ind.

[32] Guo L, Tan DC, Hui FY, Gu F, Xiao KM, Hua Y (2019). Optimization of the cellulase-ultrasonic synergistic extraction conditions of polysaccharides from *Lenzites betulina*. Chem Biodiver.

[33] Qin WD, Ma LH, Chen XH, Zhao Y (2008). Study on extraction and deprotein of polysacharide in ginger. Food Chem.

[34] Wang LW, Ou YZ, Zhang BJ, Yang Q, Wang DJ, Yu YF, Zhang J, Ma Q (2017). Deproteinization and structural analysis of polysaccharides from *Dendrobium officinale* Kimura et Migo grown in Huoshan. Food Chem.

[35] Wang YP, Wang CN, Guo MR (2019). Effects of ultrasound treatment on extraction and rheological properties of polysaccharides from *Auricularia Cornea* var. Li. Molecules.

[36] Li SF, Wang AJ, Tian GR, Liu LN, Wei SX (2018). Column chromatographic purification and monosaccharide composition of polysaccharide extracted from *Agaricus bisporus* stipe. Sci Technol Food Ind.

[37] Zhang ZP, Shen CC, Gao FL, Wei H, Ren DF, Lu J (2017). Isolation, purification and structural characterization of two novel water-soluble polysaccharides from *Anredera cordifolia*. Molecules.

[38] Wang XZ, Li F, Zhao YZ (2019). Study on ultra-high pressure extraction of *Aurea helianthustems* polysaccharides and its antioxidant activity. Cereal Oils.

[39] Venkatesan T, Choi YW, Kin YK (2019). Effect of an extraction solvent in the antioxidant quality of *Pinus densiflora* needle extract. J Pharm Analysis.

[40] Cai LL, Chen BH, Yi FL, Zou SS (2019). Optimization of extraction of polysaccharide from dandelion root by response surface methodology: structural characterization and antioxidant activity. Int J Biol Macromol.

[41] Li H (2017). Extraction, purification, characterization and antioxidant activities of polysaccharides from *Ramaria botrytis* (Pers.) Ricken. Chem Cent J.

